# Socially Enforced Nepotism: How Norms and Reputation Can Amplify Kin Altruism

**DOI:** 10.1371/journal.pone.0155596

**Published:** 2016-06-15

**Authors:** Doug Jones

**Affiliations:** Department of Anthropology, University of Utah, Salt Lake City, Utah, United States of America; Tianjin University of Technology, CHINA

## Abstract

Kin selection, which can lead organisms to behave altruistically to their genetic relatives, works differently when—as is often the case in human societies—altruism can be boosted by social pressure. Here I present a model of social norms enforced by indirect reciprocity. In the model there are many alternative stable allocations of rewards (“distributional norms”); a stable norm is stable in the sense that each player is best off following the norm if other players do the same. Stable norms vary widely in how equally they reward players with unequal abilities. In a population of mixed groups (some group members follow one norm, some follow another, and some compromise) with modest within-group coefficients of relatedness, selection within groups favors those who compromise, and selection between groups favors generous generalized reciprocity rather than balanced reciprocity. Thus evolved social norms can amplify kin altruism, giving rise to a uniquely human mode of kin-based sociality distinct from spontaneous altruism among close kin, or cooperation among non-kin.

## Introduction: Beating Hamilton’s Rule

This article presents a souped-up version of the theory of kin selection. In the standard version, an organism has a chance to provide benefit *b* to one of her kin at cost *c* to herself. Natural selection favors this altruistic act as long as Hamilton’s Rule is satisfied, *r* ⋅ *b* > *c*, where *r* is the coefficient of relatedness of recipient to donor [1]. But in the theory presented here, altruism toward kin is influenced by a wider social context, as members of a group play a game in which each player can reward others for behaving generously toward their mutual kin. I show that this can favor the evolution of high levels of altruism, in the form of unbalanced generalized reciprocity among kin, even when *r* is small.

The theory of socially enforced nepotism should be of interest to scholars in several disciplines [2, 3]. It bears on arguments among evolutionary theorists about how useful inclusive fitness theory is in accounting for social behavior, among social scientists about the relationship between rational choice and social norms, and among cultural anthropologists about how far their theories of kinship can be reconciled with evolutionary theories.

In the rest of this introduction, I summarize theoretical and empirical reasons for wanting to add a model of social norms and their enforcement to the theory of kin selection. On the theoretical side: Kin selection theory is sometimes equated with the proposition that organisms are shaped by natural selection to maximize their inclusive fitness. But this result rests on the assumption that for each actor in a group, we can separate out an independent contribution that she makes to the well-being of her kin [4]. This amounts to treating kin selection as a collection of one-person games.

However kin selection gets more complicated when there is more in the way of strategic interaction among actors. Consider a simple model which combines game theory and population genetics, the Brothers Karamazov Game [5]. The game is played among three brothers, with a coefficient of relatedness *r* = 1/2 for each pair. One brother is a potential recipient of altruism, and the other two are potential donors, so we are looking at a two-person game between the two donors regarding how they treat the third. To establish a benchmark for comparison, we begin with each of the two potential helpers deciding independently of the other one whether to help their needy sibling. In this case, the evolution of altruism is governed by Hamilton’s Rule. Each brother should help as long as his cost *c* and the needy brother’s benefit *b* satisfy 1/2 ⋅ *b* > *c*. But now consider the case where the two potential helpers adopt a strategy of *conditional nepotism*: each will provide extra help as long as the other does the same. It is tempting to analyze this package deal using inclusive fitness theory, with costs and benefits to one’s siblings counting for half as much as costs to oneself. By this accounting, with two brothers taking turns, each on his turn paying cost *c* and providing benefit *b* to the third, the rule for altruism is 2/3 ⋅ *b* > *c*. But a more careful analysis using gene and genotype frequencies gives a different answer: the condition for the evolution of altruism is *r*_*C*_ ⋅ *b* > *c*, where *r*_*C*_ = (1 + 6*p*)/(2 + 8*p*), with *p* being the frequency of the allele for conditional altruism. The conditional coefficient of relatedness *r*_*C*_ increases from 1/2 to 7/10 as *p* increases from 0 to 1. A conditional nepotism gene cannot invade a population when very rare, but can spread once it gains a foothold.

The Brothers Karamazov Game demonstrates several things. First, it shows the limitations of inclusive fitness accounting. The calculation based on the genealogical coefficient of relatedness of 1/2 between siblings doesn’t work, because the probability that the two helpers are identical-by-descent for the conditional nepotism allele is related in a complicated way to the strategies each chooses. While inclusive fitness theory can offer qualitative insights, it takes an explicit population genetic calculation to get the correct answer. Second, the model provides a proof-of-concept that a socially enforced rule can amplify kin altruism. The conditional strategy can lead each donor brother to behave *as if* he were closer kin than a brother. One way to understand this is to note that altruism toward kin is a public good. When one brother provides help, he is also giving a free boost to the inclusive fitness of the other potential helper, and vice versa. As with other public goods, more is provided when players reduce free-riding by enforcing agreed-upon rules. In this case, if we gauge kin altruism by *c*/*b*, then conditional nepotism beats individual nepotism following Hamilton’s Rule by up to 40 percent.

The Brothers Karamazov Game is perhaps the simplest possible model of socially enforced nepotism. The bulk of this article explores a more general model of an *n*-person game that differs from the Brothers Karamazov Game in several respects. First, the model here is not built around a simple two-fold division of players into those who help and those who are helped. Instead it includes players with a range of helping abilities. Second, players in the model do not rely on a simple conditional strategy to boost altruism to kin. Such a strategy (“I’ll help if you do too”) works for two helpers. But the *n*-person conditional strategy (“I’ll help if everyone else does too”) breaks down for *n* much greater than two, because it imposes an impossibly stringent unanimity requirement [6]. Instead, players rely on indirect reciprocity and honest advertising of ability to enforce norms of sharing. The model lets us explore the contrast, and the evolutionary contest, between norms of balanced reciprocity (where player *j* helps player *i* as much as *i* helps *j*) and generalized reciprocity (where *j*, concerned with her reputation among other players, helps *i* even if *i* cannot reciprocate). Many anthropologists regard generalized reciprocity, or communal sharing [7], as a hallmark of relations among kin.

This brings us to the empirical motivation for the present work. Long before evolutionary biologists devised the theory of kin selection, cultural anthropologists developed their own theory of kinship—perhaps the discipline’s single greatest contribution to the human sciences. The study of kinship is important because, as one scholar writes, “In many societies, both primitive and sophisticated, relationships to ancestors and kin have been the key relationships in the social structure; they have been the pivots on which most interaction, most claims and obligations, most loyalties and sentiments, turned” [8]. The study of kinship has resulted in a number of important findings; of special interest here is a generalization regarding the connection between kinship and altruism: “Kindred and kindness go together—two words whose common derivation expresses … one of the main principles of social life” [9]. In other words,

the central value premise associated with [the notion of kinship] is uniform. Kinship predicates the axiom of amity, the prescriptive altruism exhibited in the ethic of generosity. … [T]he rule posits … that “kinsfolk” have irresistible claims on one another’s support and consideration … simply by reason of the fact that they are kin. Kinsfolk must ideally share … and they must, ideally, do so without putting a price on what they give. [10]

The observation that people act altruistically toward their kin even when they can expect nothing in return sounds like what the theory of kin selection predicts. But there is a crucial complication: human kinship has a normative side, as implied by the words “prescriptive,” “ethic,” “rule,” and “ideally” above. How one person treats others, including her kin, is partly guided by her personal feelings toward them but also partly dictated by social conventions. This means, according to many anthropologists, that human kin altruism comes in two varieties. (Much of the evidence regarding the two varieties of nepotism comes from a large body of qualitative ethnographic studies, but the distinction has also been upheld by recent quantitative research [11, 12].) One variety (call it *individual nepotism*) is motivated by benefactors’ feelings of attachment to their beneficiaries. It is largely directed to close kin (consistent with inclusive fitness theory, since coefficients of relatedness fall off rapidly with genealogical distance). The second variety (call it *socially enforced nepotism*) is governed by conventions about how kin ought to be treated, and is motivated by social pressure and concern for one’s standing in a moral community. It can extend even to distant kin. “[T]he most convincing evidence … of kinship amity [is found in] situations where kinship is so tenuous as to be only nominal, as when persons seek out remote clansfolk … and without further ado claim and receive hospitality and protection.” [10] It is socially imposed nepotism that helps make kinship important not just for family life, but for social organization on a larger scale.

Individual nepotism looks a lot like kin altruism in other species [13]. Socially enforced nepotism is different. It depends on the moral regulation of behavior according to socially transmitted norms—something regarded by many social scientists, including some evolution-minded ones, as a distinguishing feature of our species [14–16]. Hence the model below, combining game theory and population genetics, in which the starting point is not an individual deciding on her own how much to help her kin, but a group of unequal individuals settling on and enforcing a norm determining who gets how much.

## Results

### Distributional norms, reputation, and incentives

Imagine social life as a drama [17]. Each individual plays the role(s) appropriate to her status, treating other players as directed by her role and theirs, with all players acting from a shared script. As part of the drama, real costs are paid and benefits received, so a player has an extrinsic motive to stick to the script: if she breaks character, other players react so as to leave her with lower net benefits. Here I translate this dramaturgical metaphor into a game-theoretical model in which players use indirect reciprocity and reputation to get one another to play their parts. In the model, players differ in their ability to help others (= status); they help and are helped based on their advertised abilities (= assumed roles); and each has an incentive to honestly advertise her ability by following a distributional norm (= shared script).

Consider a game among a group of *n* players. In each round of play, each player has the opportunity to help some randomly selected target players. Costs and benefits of helping are related by the production function

$$\begin{array}{c} b_{ij}=\alpha_{j}^{1-\tau }c_{ji}^{\tau } \end{array}$$ (1)

where *b*_*ij*_ is the benefit that *i* receives from *j*, *c*_*ji*_ is the cost that *j* incurs in helping *i* (both averaged over a long span of play), *α*_*j*_ is a measure of *j*’s productive ability, and *τ* generates diminishing marginal returns to helping, with *b*_*ij*_, *c*_*ji*_, and *α*_*j*_ ≥ 0, and 0 < *τ* < 1.

We are interested in indirect reciprocity, where player *j* produces benefits for player *i* based on the benefits *i* has produced for other players in earlier rounds of play [18–25]. Who produces how much for whom—the value of *b*_*ij*_—depends on *distributional norms* and *reputations*. Let’s assume initially that members of a group agree on a single distributional norm, call it *A*, defined by a function *b*^*A*^[*x*, *y*] over *x* ≥ 0, *y* ≥ 0. This function dictates how much benefit a player with ability *x* ought to get from a player with ability *y*. If *i* and *j* have abilities *α*_*i*_ and *α*_*j*_, and *j* follows the norm, then *b*_*ij*_ = *b*^*A*^[*α*_*i*_, *α*_*j*_]. Suppose however that *j* cannot assess *i*’s ability directly, but must act according to an ascribed ability, or reputation, for *i*. To achieve this, the function *b*^*A*^ must made to operate in two directions, determining both the benefits *j* produces for other players *and* the reputation others assign her. First, if *j* knows her own ability, *α*_*j*_, and ascribes ability *s*_*i*_ to player *i*, she can reward *i* according to *b*_*ij*_ = *b*^*A*^[*s*_*i*_, *α*_*j*_]. Second, if some player *k* knows what benefits *j* has produced for other players, and has ascribed abilities for them, she can work backward to assign a new reputation, *s*_*j*_, to *j*, where *s*_*j*_ is implicitly given by *b*^*A*^ (as spelled out below). This means that reputation is defined recursively: the reputation of each player depends on the benefits she bestows on others and on their reputations. The result, at equilibrium, is that reputation equals ability (*s*_*j*_ = *α*_*j*_) and players receive benefits according to *b*_*ij*_ = *b*^*A*^[*α*_*i*_, *α*_*j*_] and pay costs according to $c_{ji}=\alpha_{j}^{-\frac{1-\tau }{\tau }}b^{A}{\left[\alpha_{i},\alpha_{j}\right]}^{1/\tau }$, for all *i* and *j*.

**Fig 1:**
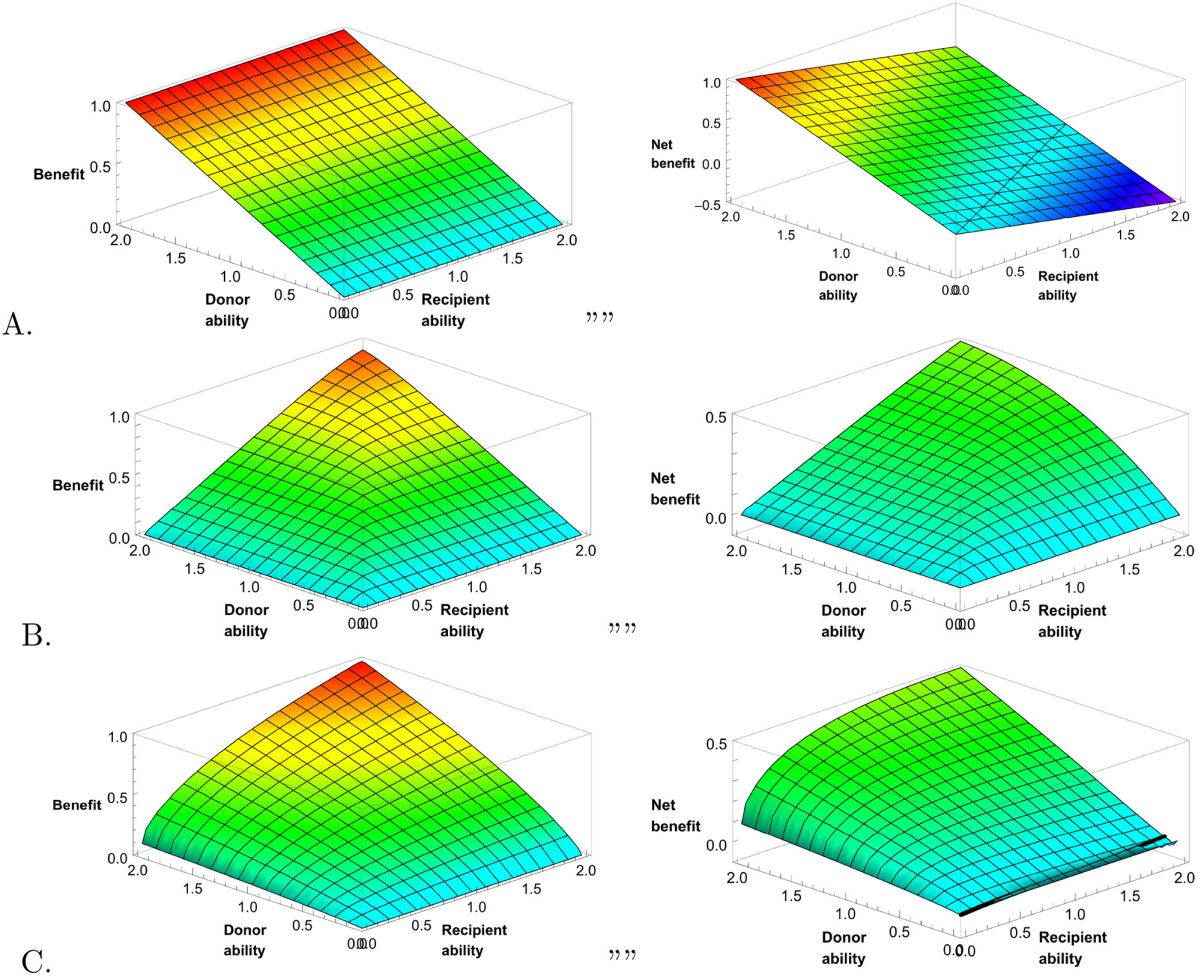
Three distributional norms. How benefits should be distributed as a function of recipient (*x*) and donor (*y*) ability, according to norms of (A) Pure Altruism, (B) Almost Balanced Reciprocity, and (C) Generalized Reciprocity. On left, benefits, *b*[*x*, *y*]; on right, recipient net benefits, *b*[*x*, *y*] − *c*[*x*, *y*]. Colors scale with benefits or net benefits, 1.0 is red, 0.0 is light blue. Black lines show where net benefits equal zero.

In principle, any norm with non-negative *b*^*A*^[*x*, *y*] is allowed, including a norm of pure altruism, “from each according to his ability, to each according to his need” (Fig 1A). But here we are especially interested in the class of incentive-compatible stable norms, which motivate each self-interested player to honestly advertise her ability by following the norm, provided others do likewise. A stable norm and associated reputation function must satisfy several conditions.

(i) *Positive net benefits*. For each active player *j*, summed benefits minus summed costs averaged over many rounds of play must be greater than zero.

$$\begin{array}{c} \sum_{k}b^{A}\left[\alpha_{j},\alpha_{k}\right]-\alpha_{j}^{-\frac{1-\tau }{\tau }}\sum_{i}b^{A}{\left[\alpha_{i},\alpha_{j}\right]}^{1/\tau }>0 \end{array}$$ (2)

(ii) *Penalty for impersonation*. For each active player *j*, her summed net benefits must be greater if she honestly advertises her ability, *α*_*j*_, than if she “impersonates” a player of lesser or greater ability, *y*:

$$\begin{array}{c} \sum_{k}b^{A}\left[\alpha_{j},\alpha_{k}\right]-\alpha_{j}^{-\frac{1-\tau }{\tau }}\sum_{i}b^{A}{\left[\alpha_{i},\alpha_{j}\right]}^{1/\tau }\\ >\sum_{k}b^{A}\left[y,\alpha_{k}\right]-\alpha_{j}^{-\frac{1-\tau }{\tau }}\sum_{i}b^{A}{\left[\alpha_{i},y\right]}^{1/\tau } \end{array}$$ (3)

for *y* ≠ *α*_*j*_. So summed net benefits must increase, neither too slowly nor too quickly, with donor ability.

(iii) *Penalty for inconsistency*. Player *j* might be less generous to some players and more generous to others than is consistent with honestly advertising her ability *a*_*j*_ or impersonating a player with ability *y*: there might not be any one *y* such that *b*_*ij*_ = *b*^*A*^[*s*_*i*_, *y*] for all *i*. In this case, *j* is assigned an intermediate weighted average reputation, *s*_*j*_, chosen so that *b*_*ij*_ < *b*^*A*^[*s*_*i*_, *s*_*j*_] for some *i* and *b*_*ij*_ > *b*^*A*^[*s*_*i*_, *s*_*j*_] for other *i*. The aim is not to make *s*_*j*_ an accurate estimate of *α*_*j*_, but to penalize *j* for behaving inconsistently. For example, let

$$\begin{array}{c} 0=\sum_{i}\left(b_{ij}^{1/\tau }-b^{A}{\left[s_{i},s_{j}\right]}^{1/\tau }\right)\omega^{\left(b_{ij}^{1/\tau }-b^{A}{\left[s_{i},s_{j}\right]}^{1/\tau }\right)} \end{array}$$ (4)

This gives *s*_*j*_ as an implicit function of the benefits *j* produces, *b*_*ij*_, and the reputations of her beneficiaries, *s*_*i*_. Suppose *ω* = 1. Then the *ω* term drops out, and *j*’s actual cost, $\alpha_{j}^{-\frac{1-\tau }{\tau }}\sum_{i}b_{ij}^{1/\tau }$, is equal to the cost, $\alpha_{j}^{-\frac{1-\tau }{\tau }}\sum_{i}b^{A}{\left[s_{i},s_{j}\right]}^{1/\tau }$, she would pay if she consistently impersonated a player with ability *s*_*j*_. So inconsistent play gets her the same reputation, for the same cost, as consistent play. But suppose instead that 0 < *ω* < 1. Then *j* pays the same cost as before, but her reputation is now lower, because subpar *b*_*ij*_’s are more heavily weighted, and reduce *s*_*j*_ more than excessive *b*_*ij*_’s increase it. So we make 0 < *ω* < 1: either *j* is consistent, and gets the appropriate *s*_*j*_ for honest advertising or consistent impersonation, or *j* is inconsistent, and pays a reputational penalty.

With a distributional norm and a reputation function that satisfy these conditions, consistent honest advertising is the best strategy for each player, as long as others follow the norm. So the honest advertising constraints set limits on what distributional norms are stable among self-interested players—for example, the norm matching the payoffs under pure altruism is *not* stable. But within these limits, there is a wide range of stable norms, including the two below, which differ in how well they treat weak players, and are meant to capture the anthropologist’s distinction between balanced and generalized reciprocity.

### Almost Balanced Reciprocity

One family of distributional norms assigns equal payoffs in both directions, *b*^*A*^[*x*, *y*] = *b*^*A*^[*y*, *x*] for all *x* and *y*. Players get only as much they give, and “there ain’t no such thing as a free lunch.” We can pick one member of this family by allowing the weaker player to set *b*^*A*^[*x*, *y*] = *b*^*A*^[*y*, *x*] to her preferred value, $\tau^{-\frac{1-\tau }{\tau }}x$ for *x* ≤ *y*. This norm is stable under direct reciprocity, and also under indirect reciprocity where players estimate reputations according to Condition 4.

**Fig 2:**
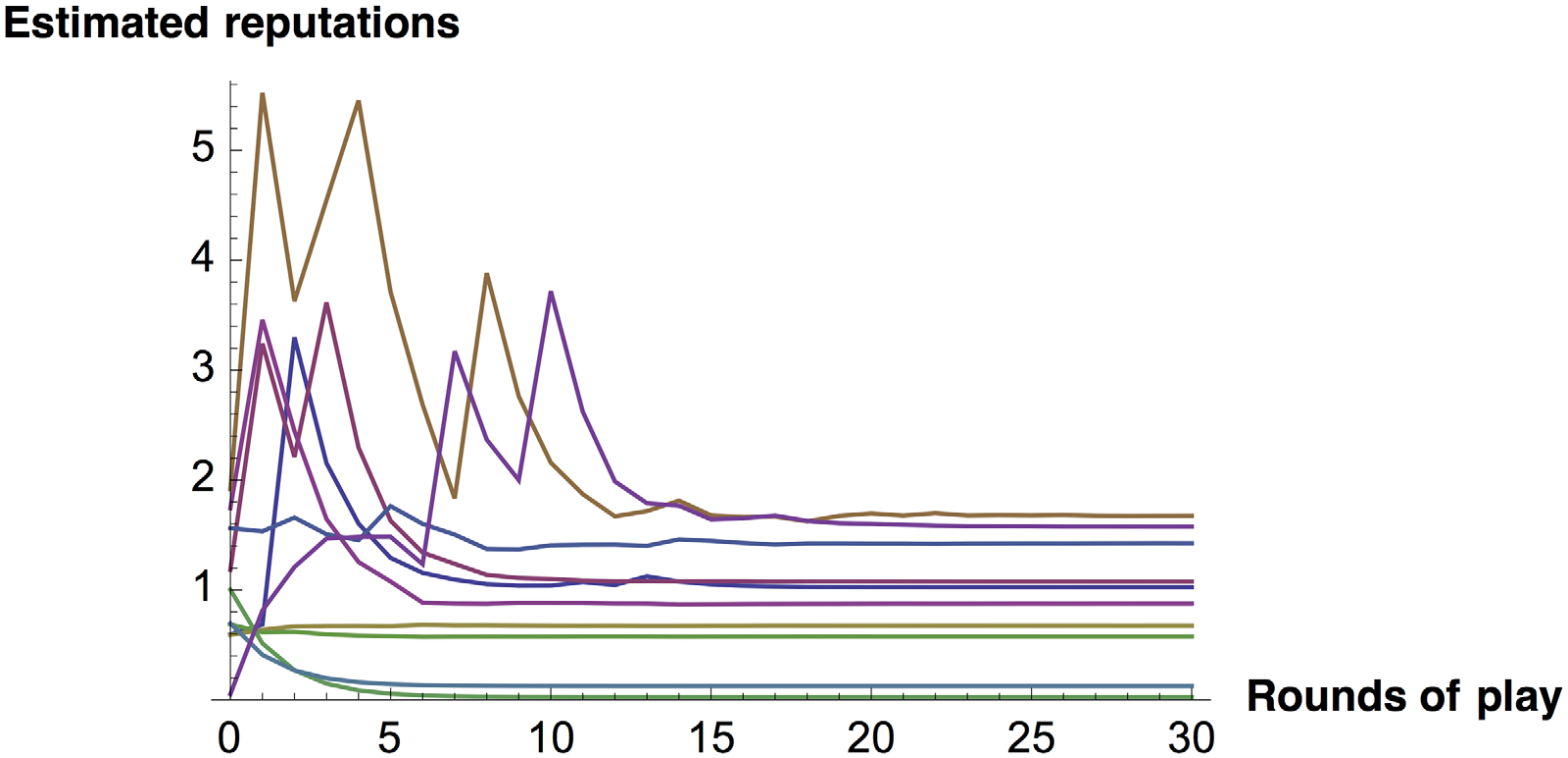
Reaching equilibrium with Almost Balanced Reciprocity. In a group of 40 players, each player *j* produces benefits for a random 5 players *i* in each round, following *b*^*B*^ and beginning in round 1 using random private estimates of *s*_*i*_. For each *j*, every other player *k* calculates a revised *s*_*j*_ of her own, after observing a random 3 of *j*’s 5 random acts of kindness, by plugging the 3 *b*_*ij*_’s and her 3 unrevised *s*_*i*_’s into the implicit function given by Condition 4. For each *j*, player *k* then averages her revised and unrevised *s*_*j*_’s to generate new *s*_*j*_’s that will determine how much she helps a random 5 players in the next round. Chart shows a typical result for a random player’s private estimates of *s*_*j*_’s for 10 other random players *j*: her *s*_*j*_’s converge on *α*_*j*_’s. (Generalized Reciprocity gives similar results.)

There’s a hitch, however. Exact balanced reciprocity, direct or indirect, is not *dynamically* stable. If players start somewhere other than at equilibrium, and estimate one another’s abilities based on past play, then reputations and benefits produced don’t converge on the equilibrium. Even small perturbations are destabilizing. The underlying problem is familiar from the Iterated Prisoner’s Dilemma game: two tit-for-tat players don’t get to the cooperative equilibrium if they aren’t already there. In the prisoner’s dilemma case, this can be fixed with a forgiving amendment to the tit-for-tat strategy [26]. In the present case, the analogous fix is to set *b*^*A*^[*x*, *y*] slightly higher than *b*^*A*^[*y*, *x*] for *x* < *y*, with the difference adjusted to satisfy Condition 3. We call the resulting stable distributional norm Almost Balanced Reciprocity, or B, with defining function *b*^*B*^ (Figs 1B and 2).

### Generalized Reciprocity

With Almost Balanced Reciprocity, a strong player could provide a weak player with greater benefits at little added cost to herself, but doesn’t do so because it would be too costly for the weak player to (almost) match the extra benefits. Discarding the principle that exchanges must balance allows for a more even distribution of benefits and greater total net benefits. Consider the norm that maximizes group net benefits, subject to the honest advertising constraints. With this norm, a strong player gives substantial help to weak players, even entirely helpless players with *α* = 0, in order to uphold her reputation and be rewarded by other strong players. This is socially enforced altruism. It is less generous than pure altruism, because strong players need to do better than weak players to encourage honest advertising, but more generous than Almost Balanced Reciprocity.

An even more generous distribution is possible if we maximize total net benefits, but allow *j* some knowledge, not just of the benefits *i* produces, but of the costs *i* pays. In this case, a weak *i* helping others at some cost to herself can receive more credit than a stronger player providing the same benefits at less cost [27], making it possible to enforce more generosity toward weak players without motivating shirking among strong players. Here we use a version of a distributional norm with some knowledge of costs, calling it Generalized Reciprocity, or G, with defining function *b*^*G*^ (Fig 1C).

### Mixed groups, compromise, and kin selection

Either norm, Almost Balanced Reciprocity (B) or Generalized Reciprocity (G), is stable if everyone agrees to it. But what happens if players don’t agree? Evolutionary considerations can help answer this question.

**Fig 3:**
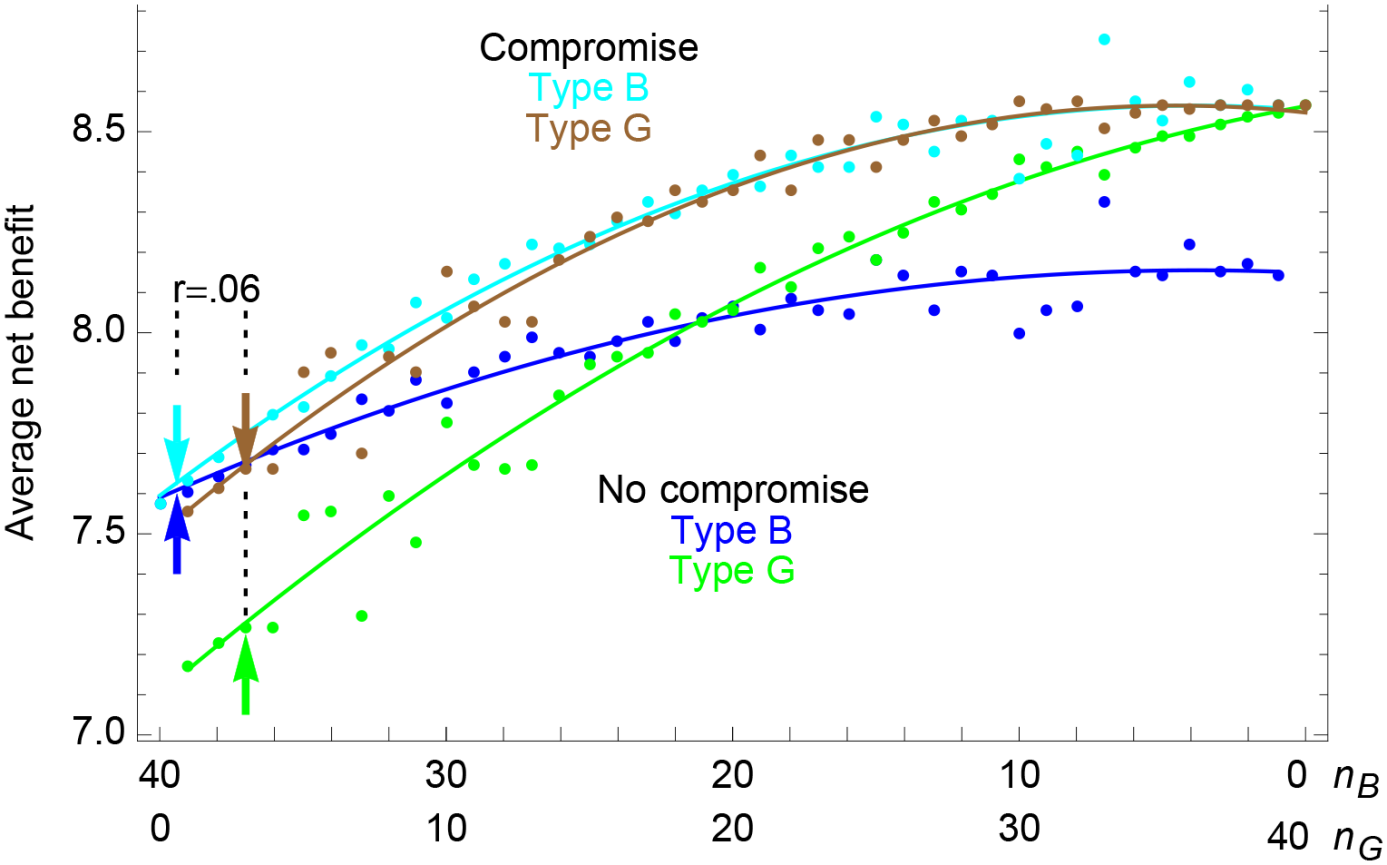
Mixed groups. Average net benefits in mixed groups of 40 players, with *n*_*B*_ playing Almost Balanced Reciprocity and *n*_*G*_ playing Generalized Reciprocity—or with the two types compromising on an intermediate norm—based on random sampling of players with differing abilities. Lines show quadratic best fits to points. Arrows show that with a modest coefficient of relatedness (*r* = .06, suggested by [5, 28]) and no compromise, a rare type G does worse than a common type B, but with compromise, a rare type G does better.

Consider a mixed group, in which some players follow norm B and some follow G. In such a group, each player *j* can be assigned two reputations, $s_{j}^{B}$ and $s_{j}^{G}$, depending on the benefits she produces and the reputations, $s_{i}^{B}$ and $s_{i}^{G}$, of her beneficiaries. We can numerically calculate an equilibrium, at which type B players reward other players according to their *s*^*B*^ reputations and type G players reward according to *s*^*G*^ (Table 1). We can further calculate the mean net benefits accruing to each type. Simulations show that in groups with a given frequency of types B and G, the more common type usually gets greater mean net benefits; but in a collection of groups with different frequencies of B and G, both types usually get greater mean net benefits in those groups where B is less frequent and G more frequent (Fig 3, lower portion).

**Table 1:** Reputation in a mixed group. Equilibrium reputations, *s*^*B*^ and *s*^*G*^, calculated for a mixed group of 40 players of varying ability (*α*). A random 20 players follow norm B and 20 follow G; results are given for three representative players of each type. Players accurately assess abilities of those of their own type, but under- or overestimate abilities of those of unlike type.)

Type	*α*	*s*^*B*^	*s*^*G*^
B	1.925	1.925	1.156
B	1.025	1.025	.934
B	.325	.325	.229
G	1.725	1.958	1.725
G	.925	.968	.925
G	.125	.178	.125

Now consider a large population of groups, each of size *n* and average within-group relatedness *r*, where the overall frequency of type B in the population is *p*_*B*_. On average, a B player finds herself in a group where type B numbers 1 + (*n*−1)(*r* + *p*_*B*_ − *r* ⋅ *p*_*B*_), and a G player finds herself in a group where type B numbers (*n*−1)(*p*_*B*_ − *r* ⋅ *p*_*B*_) (lower arrows in Fig 3). If *n* is not very small, and *r* is not very large, either number is close to *n* ⋅ *p*_*B*_. If net benefits are proportional to increments in fitness, then the common type (away from the equal payoff crossover point) is expected to have higher fitness in most groups. Selection within groups, favoring the common type, is stronger than selection between groups, favoring G. Whichever distributional norm starts out at high frequency goes to fixation.

Yet the survival of the commonest is not the end of the evolutionary story. The equilibrium where some group members play B and others G is not a Nash equilibrium. Individuals of either type can do better if they don’t stick to their preferred strategy, but adapt to the presence of the other type. Such accommodation within a mixed group can take various forms.

First, all players can compromise on an intermediate strategy. A compromise distributional norm (call it W) can be defined by a parameter *w*_0_, with 0 < *w*_0_ < 1, such that the summed net benefit received by a player with ability *y* is a weighted mean of the summed net benefits received by a *y* player under norms B and G, with weights *w*_0_ and 1−*w*_0_. For a given *w*_0_, a benefit function *b*^*W*^ can be defined such that the corresponding norm W is stable, giving each player an incentive to honestly advertise her abilities as long as others follow the norm. Here we allow each group to settle on the *w*_0_ for which *b*^*W*^^1/*τ*^ gives a least-squares best fit to $b_{ij}^{1/\tau }$ for type B and type G players.

When players compromise like this, simulations give different results than when they don’t (Fig 3). Both types do better when they settle on an intermediate norm W. Furthermore, among compromising groups with a given frequency of types B and G, B usually does slightly better than G. This is because strong players have more influence on the value of *w*_0_ than weak players. In groups where more of the strong players are type B, the B’s “vote” greater net benefits for the strongest players, themselves; where more of the strong players are type G, the G’s “vote” greater net benefits for the average player. But G can still prevail: with positive within-group relatedness, type G players are more likely to find themselves in groups with extra type G players (upper arrows in Fig 3). Strong G players’ support for higher mean net benefits in these groups can give G a greater average fitness.

**Fig 4:**
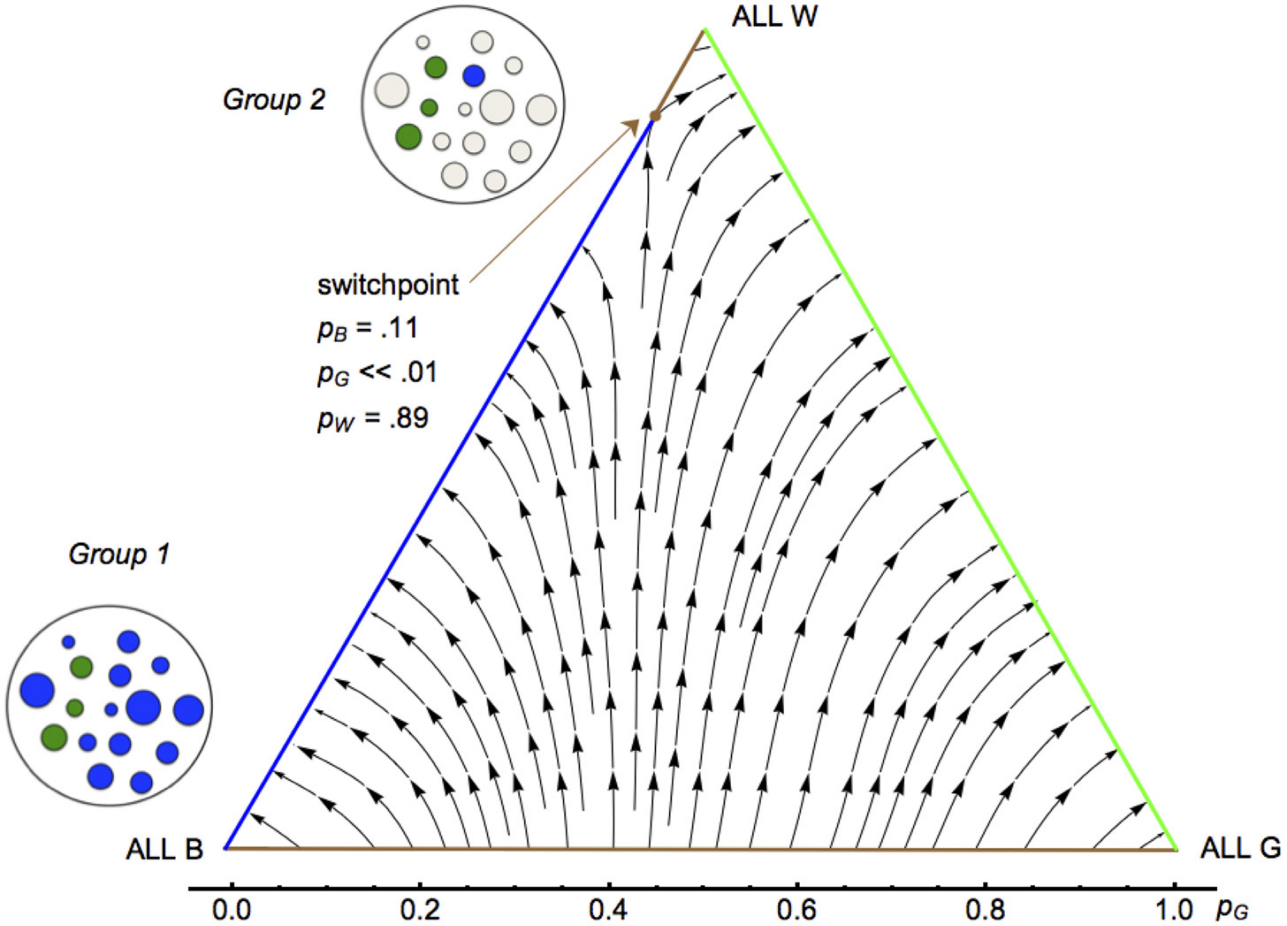
Three types. Stream plot of selection vectors for populations with fractions *p*_*B*_, *p*_*G*_, and *p*_*W*_ playing Almost Balanced Reciprocity, Generalized Reciprocity, and a Weighted Compromise. Fitnesses are proportional to net benefits, averaged over random samples of mixed groups of 40 players with coefficient of relatedness *r* = .06. Blue line shows a neutrally stable mix of B and W players, green line a neutrally stable mix of G and W players. At switchpoint, the mixture of B and W is unstable: population leaves B/W equilibrium for G/W, never to return. *Group 1*: Typical group composition experienced by a rare G player and her relatives when type W is absent. Type G (green circles; size stands for ability) is outnumbered by type B (blue circles). *Group 2*: Typical group composition experienced by a rare G player and her relatives when type W is common. G player and her G relatives outnumber type B; type W players (white circles) skew their play toward Generalized Reciprocity, amplifying small *r*.

In an alternative form of compromise, type B and G players persist in playing their respective strategies, while a third type of player, type W, adopts the compromise strategy corresponding to the group’s least-squares *w*_0_. Experimentation with a range of parameter values shows that in groups where B and G players refuse to compromise, type W—a moral chameleon who takes on the average color of her surroundings—generally does better (Fig 4). Once type W reaches high frequency, selection changes, and the rarest type is no longer at such a disadvantage. In a population with a very common type W, rare type G, less rare type B, and moderate within-group relatedness, a type G player may often find herself in a group containing many type W’s, several type G kin, and fewer type B’s. Such a group will lean toward enforcing a generous, G-weighted norm, to the advantage of type G.

So combining a small measure of principled rule-following with a large measure of self-interested compromise opens up new space for kin selection. When types B and G compromise on norm W, variance within groups decreases. When B and G don’t compromise and the compromising type W becomes more frequent, variance between groups increases. Either way, compromise strengthens selection between groups relative to selection within them. With socially enforced altruism, a little relatedness can go a long way.

## Discussion

A big motive for developing the theory of kin selection was the problem of *altruism*: Why does one organism sometimes help another when that help will not be reciprocated? The analogous motive for developing a theory of socially enforced nepotism is the problem of *distribution*: How are the gains from cooperation distributed? [29] Why does social exchange sometimes shade into uni-directional generosity—balanced reciprocity into generalized reciprocity?

Previous theory [19] and evidence [30, 31] show that indirect reciprocity can motivate cooperation in the form of contributions to public goods. The model developed here goes further. It is simultaneously about motivating cooperation *and* about distributing the gains from cooperation among players of unequal abilities. Kinship is part of the story because even modest coefficients of relatedness—too low to motivate much individual nepotism—can turn the problem of distribution from a constant sum game into a public goods game: all are better off evolutionarily if strong players give up potential net benefits to help weak players. (A rule of thumb: if *r* ≫ 1/*n*, then a player can expect that other group members carry more copies of her genes than she does, and she should “vote” for the norm that is best for the average player, even if it’s not best for her. [5])

The work here extends earlier findings that indirect reciprocity depends on reputation and reputation must be defined recursively, so that each player’s reputation depends both on the benefits she provides and on the reputations of the beneficiaries. In the model here, reputation is an index of ability, and, like ability, can vary continuously. So the model faces the problem of assigning a numerical reputation to each player based on the reputations of other players. This is more complicated than simply choosing to cooperate or not based on whether the other player is in good or bad standing. It is close to the problem of designing a page-ranking algorithm that ranks a collection of web pages by assigning a score to each page based on the scores of the pages that link to it [32]. Some of the same issues arise in both cases: for example, whether a given distributional norm or page-ranking algorithm is dynamically stable and converges on a consistent result.

The model must satisfy a further set of conditions. We require that a distributional norm be enforceable (or *incentive compatible*): a player of whatever ability should do better sticking to the norm, rather than impersonating a stronger or weaker player or playing inconsistently, as long as other players do the same. All these conditions rule out many distributional norms. For example, strict balanced reciprocity is dynamically unstable, and pure altruism is unenforceable. But the conditions also allow a wide range of distributional norms with very different distributions of benefits, including the two we concentrate on here, Almost Balanced Reciprocity and Generalized Reciprocity.

One way to think about how reputation works in enforcing one norm or another is to compare it to another sort of currency, money. Either reputation or money can be used for indirect reciprocity: one person helps another in order to gain reputational or monetary credit, not for its own sake, but to “buy” favors from third parties. Yet a moral economy based on reputation is different from a money economy. Money makes indirect reciprocity work by reducing it to direct reciprocity, to dyadic money-for-benefits trades. A money-based economy can scale up indefinitely, coordinating exchanges among millions of strangers. But it has a hard time harnessing these transactions to a common social purpose; money-based economies normally rely on auxiliary political institutions to produce desired public goods. Reputation, by contrast, doesn’t scale up so easily, and works most readily in face-to-face groups, where reputations can be a matter of common knowledge—known to each, and known to be known to each [33]. Because the community decides, based on shared values, how much credit one player gains for helping another, reputation is more easily enlisted for social purposes.

How one norm or another is selected from among those allowed is a further question. A feature of many models of social enforcement through indirect reciprocity is that once a norm is in place it is locked in, and resistant to change. This makes it hard to explain how groups might shift from a merely adequate norm to a better one. In the present model, lock-in occurs in the simplest case. With just two norms competing in a population of groups, the winner is the norm that starts out at higher frequency, not necessarily the best. But the analysis here also shows how lock-in can be avoided: in mixed groups, those who compromise on an intermediate norm, rather than sticking to either pure norm, do better. Compromise, which is favored by within-group selection, also acts as a catalyst, lowering the barrier to a transition between norms and facilitating between-group selection in favor of rare pro-social norms, like Generalized Reciprocity.

Like most models of human sociality, the present model of socially enforced nepotism concentrates on just one part of the process of social evolution, and treats other parts more cursorily. In this spirit, we make the simplest possible assumption about how group membership translates into fitness: groups that produce more offspring make a proportionately greater contribution to the whole population in the next generation. This is multi-level selection, but not group selection in the strong sense—differential multiplication and extinction of groups—which would require additional assumptions about population processes. We also implicitly adopt a *weak selection* assumption: we ignore the effects of selection between the time a gene is present in an ancestor and the time it acts in her descendants, which can make genealogical relatedness a poor indicator of identity by descent for genes under strong selection. This assumption is probably roughly correct for groups on the scale of local groups, lineages, and clans, but not on the scale of tribes and ethnic groups.

These simple assumptions about population dynamics and population genetics might be revised in later work. But here the focus has been on an aspect of sociality that cultural anthropologists past and present have highlighted: norms and roles in relation to kin altruism. Some anthropologists argue that human kinship, insofar as it is socially enforced, is divorced from biology. The argument here, on the contrary, is that kinship is uniquely elaborate and important in our species because norms that push people to treat distant kin like close kin have been favored by natural selection.

## Supporting Information

S1 File Supporting information. Further information about mathematical results and numerical simulations, including references to S1 and S2 Figs.
(PDF)

S1 Fig Not reaching equilibrium with exact balanced reciprocity. As in Fig 2, but with exact (not almost) balanced reciprocity. Estimated reputations do not converge on *α*_*j*_. (Many estimated reputations are wildly exaggerated, but adding a ceiling on estimates does not result in convergence.)
(PDF)

S2 Fig Weights for a compromise norm. For different values of *w*_0_, this shows how a player with ability *α*_*j*_ should weight her costs between *b*^*B*^ and *b*^*G*^ to achieve a compromise distributional norm satisfying Condition 13 for stability (See S1 File).
(PDF)
