# Supplementary material for: Socially Enforced Nepotism: How Norms and Reputation Can Amplify Kin Altruism
Source: PLoS One. 2016 Jun 15;11(6):e0155596. doi: 10.1371/journal.pone.0155596 (PMC4909296; doi:10.1371/journal.pone.0155596)
Supplement: S1 File — Further information about mathematical results and numerical simulations, including references to S1 and S2 Figs. (PDF) [file pone.0155596.s001.pdf]

Supporting information

Caption for Supporting information
Supporting information. Further information about mathematical results and numerical simulations, including references to Figs. S1 and S2.

Caption for S1 Fig
Not reaching equilibrium with exact balanced reciprocity. As in Figure 2, but with exact (not almost) balanced reciprocity. Estimated reputations do not converge on α_j. (Many estimated reputations are wildly exaggerated, but adding a ceiling on estimates does not result in convergence.)

Caption for S2 Fig
Weights for a compromise norm. For different values of w_0, this shows how a player with ability α_j should weight her costs between b^B and b^G to achieve a compromise distributional norm satisfying Condition 13 for stability.

Supporting Information

Materials and Methods
S1 and S2 Figs

The computer code used in preparing this article, in the form of Mathematica notebooks, is stored on the Open Science Framework website (osf.io) with identifiers DOI 10.17605/OSF.IO/MXZ8H / ARK c7605/osf.io/mxz8h

1 Numerical results

Numerical results presented here (Figs. 1,3,4, Table 1) are based as applicable on $\tau = .5$, $\omega = .1$, $\gamma = .5$, and a standard group of size $n = 40$ with abilities a_j evenly spaced between .025 and 1.975 at intervals of .05. Mixed groups of composition n_B and n_G, or n_B, n_G, and n_W, were generated by random sampling without replacement of players of varying abilities from the standard group. For all sums, $b_{jj} = c_{jj} = 0$ for all j. Annotated versions of code (Mathematica 9.0.1.0) and results of simulations are available on request.

To estimate average net benefits for mixed groups with differing numbers of types B and G, or B, G, and W, I selected appropriate numbers of players of differing abilities through a combination of (a) random sampling near the centers of the distributions, and (b) stratified sampling and oversampling near the ends, sides, and corners of the distributions, to reduce sampling error.

Specifically:

For mixed groups with types B and G (Fig. 3), I calculated average net benefits for each type, with and without compromise,

(a) at the center, for 31 combinations of n_B and $n_G = 40 - n_B$, with n_B and n_G both greater than or equal to 5, 32 random samples for each combination, and

(b) at the ends of the distribution, for 8 combinations with n_B or n_G between 1 and 4 inclusive, stratified sampling and oversampling of the rare type for each combination (details available on request).

For mixed groups with types B, G, and W (Fig. 4), I calculated average net benefits for each type,

(a) at the center, for 435 combinations of n_B, n_G, and $n_W = 40 - n_B - n_G$ with n_B, n_G, and n_W all greater than or equal to 4, 16 random samples for each combination, and

(b) along the sides, for 279 combinations, and at corners, for 27 combinations, of n_B, n_G, and/or n_W between 1 and 3 inclusive, stratified sampling and oversampling of the rare type(s) for each combination (details available on request).

I assume that heterozygotes play either of the corresponding homozygote strategies with probability 1/2.

2 Reputation as a function of reputation

In this model, as in previous models of indirect reciprocity, the reputation of a player depends on the reputations of those she helps. Here's why reputation has to be recursive:

Consider four sorts of strategy player j might adopt.

(A1) *Help no one.* For all i, $b_{ij} = 0$.

(A2) *Help everyone equally.* Benefits are equal for all i, depending on α_j. (For an altruistic j, who rates her costs and others' benefits equally, this would mean $b_{ij} = \tau^{-\frac{\tau}{1-\tau}} \alpha_j$.)

(A3) *Help those who help others.* Benefits for i are conditional on the summed benefits i produces for others and on j's ability, α_j, according to some function b^{cond}:

$$b_{ij} = b^{cond}[t_i, \alpha_j] \tag{5}$$

where $t_i = \sum_h b_{hi}$. A suitable b^{cond} will satisfy the honest advertising constraints (Conditions 2 and 3), with $b^{cond}[t_i, \alpha_j]$ replacing $b^A[\alpha_i, \alpha_j]$.

(A4) *Help according to reputation. Let those who help according to reputation have greater reputations.* Benefits for i depend on i's reputation – which depends on the benefits i produces for others and on their reputations – according to some function b^A satisfying Conditions 2 and 3, with reputation implicitly defined by an equation like Condition 4.

Throughout this article I assume low coefficients of relatedness and treat players as self-interested, in order to isolate the contribution of social enforcement to altruism. For self-interested players the following holds: An A1 player can invade a group in which others play A2, but cannot invade a group in which others play A3. However, if players' abilities differ, any version of A3 can be invaded by a version of A2, as follows: let $t_j = \sum_i b_{ij}$ be the total benefits produced by player j following Condition 5, then construct a version of A2 that has j producing the same total t_j, but with equal b_{ij} for all i. Because benefits are subject to increasing marginal costs, this undiscriminating beneficence provides the same summed benefits to recipients and secures the same reputation, t_j, for j, at less cost than the discriminating strategy A3.

In other words, b^{cond}, which rewards players who reward other players, is unstable because it favors a player who gives the same total benefits to other players, but plays inconsistently with b^{cond}. If abilities differ, stability requires that one player's reputation be a function of the reputations of her beneficiaries. A4 can solve both the first-order problem of policing non-cooperation, and the second-order problem of policing the police.

A similar result is found in other models of indirect reciprocity [3–7]. In a game involving many rounds of Prisoner's Dilemma, with partners switched on each round and players knowing one another's histories, a conditional strategy – cooperate with players who cooperated with their partners on the previous round(s) ("image-scoring" [8], equivalent to A3 above) – is stable. But this stability is lost if players sometimes make mistakes. A stable strategy in this case requires that a player cooperate or not with other players based on their "standing," where a player loses her good standing if she fails to cooperate with another player in good standing (equivalent to A4 above).

In summary, if nobody makes mistakes and all players have equal abilities, a strategy of "Help those who help others" is stable. But any heterogeneity among players destabilizes this strategy. Instead, a stable strategy is one in which the reputation of each player depends on the reputations of those she helps. In previous models, heterogeneity among players resulted from random errors. In the present model, players assign reputations and produce benefits based on long run average behavior; errors are assumed to average out over the long run, and to be folded into players' α's. Destabilizing heterogeneity results from unequal α's. (This conclusion depends on players following rules out of self-interest, and might change if some non-socially-enforced weak altruism based on distant kinship were allowed.)

3 Honest advertising: continuous version

Suppose y_2 lies between y_1 and y_3. It can be shown that if a player with ability y_1 is worse off impersonating a player with ability y_2, and a player with ability y_2 is worse off impersonating a player with ability y_3, then a player with ability y_1 is worse off impersonating a player with ability y_3. This means that if a distributional norm

penalizes false advertising in the neighborhood of each y, it penalizes false advertising globally, so we can write a continuous version of the honest advertising Condition 3. If player j, with ability a_j, impersonates a player with ability y, she receives benefits appropriate to y, $\sum_k b^A[y, a_k]$, while paying costs $\alpha_j^{-\frac{1-\tau}{\tau}} \sum_i b^A[a_i, y]^{1/\tau}$. She has an incentive to honestly advertise her ability if her net benefits are maximized at $y = a_j$, which implies

$$0 = \sum_k \frac{\partial b^A[y, \alpha_k]}{\partial y} - \frac{1}{\tau}\alpha_j^{-\frac{1-\tau}{\tau}} \sum_i \left(b^A[\alpha_i, y]^{\frac{1-\tau}{\tau}} \cdot \frac{\partial b^A[\alpha_k, y]}{\partial y} \right) \tag{6}$$

evaluated at $y = a_j$.

4 Reputation for inconsistent players

Here I elaborate on the definition of reputation given by Condition 4. For player j of ability a_j, the cost, summed over i, of bestowing benefits b_{ij}, is $\alpha_j^{-\frac{1-\tau}{\tau}} \sum_i b_{ij}^{1/\tau}$. The cost of "impersonating" a player of ability s_j is $\alpha_j^{-\frac{1-\tau}{\tau}} \sum_i b^A[s_i, s_j]^{1/\tau}$. Equating these sums and dividing by $\alpha_j^{-\frac{1-\tau}{\tau}}$ gives $0 = \sum_i (b_{ij}^{1/\tau} - b^A[s_i, s_j]^{1/\tau})$. If other players observe various b_{ij}, and assign j the reputation s_j implicitly given by this equation, and reward j accordingly, then her costs and benefits will be the same as if she consistently impersonated a player of reputation s_j. Call this j's unweighted reputation.

Suppose we weight each term in this sum by $\omega^{(b_{ij}{}^{1/\tau} - b^A[s_i,s_j]^{1/\tau})}$, as in Condition 4, with $0 < \omega < 1$. This weight is greater than 1 for $b_{ij}{}^{1/\tau} - b^A[s_i, s_j]^{1/\tau} < 0$, and less than 1 for $b_{ij}{}^{1/\tau} - b^A[s_i, s_j]^{1/\tau} > 0$. With subpar values of b_{ij} weighted more heavily, s_j must be shifted down to satisfy Condition 4. Call this j's weighted reputation. In this case, an inconsistent impersonator pays the costs of her unweighted reputation but only gets the benefits of a lower, weighted reputation.

5 Almost Balanced Reciprocity

With exact balanced reciprocity, $b^A[x, y] = b^A[y, x]$. In the version used here, the weaker of two players sets her marginal benefits equal to her marginal costs, giving $b^A[x, y] = b^A[y, x] = \tau^{-\frac{1-\tau}{\tau}} x$ for $x \leq y$. In a more exploitative version of exact balanced reciprocity, the weaker player could be forced into more costly, just-barely-profitable exchanges.

Here I consider the problem of dynamic instability for exact balanced reciprocity, and the solution, Almost Balanced Reciprocity. To understand why a group doesn't converge on the equilibrium if it follows the norm defined by b^A above, take the simpler case of direct reciprocity with two players and repeated rounds of play, with benefits, costs, and abilities as in Condition 1. Suppose players 1 and 2 have abilities $a_1 > 1$ and $a_2 > 1$, and start out estimating each other's abilities as .6 and .9. On the first round of play, they give each other $\{b_{12}, b_{21}\} = \{\tau^{-\frac{1-\tau}{\tau}}.9, \tau^{-\frac{1-\tau}{\tau}}.6\}$. Each player revises her estimate of the other's ability and on the next round they play $\{\tau^{-\frac{1-\tau}{\tau}}.6, \tau^{-\frac{1-\tau}{\tau}}.9\}$. On the round after they play $\{\tau^{-\frac{1-\tau}{\tau}}.9, \tau^{-\frac{1-\tau}{\tau}}.6\}$. These oscillations are equivalent to the alternations between cooperation and defection among tit-for-tat players observed in the Iterated Prisoner's Dilemma game with occasional errors. If the players base estimates of each other's abilities on an average of many rounds of play rather than just the last, the oscillations are dampened but there is no tendency to converge on the cooperative equilibrium. But with Almost Balanced Reciprocity, where $b^B[x, y]$ is slightly greater

than $b^B[y,x]$ for $x < y$, the two players each give one another slightly more than they expect to get in return on each round, until they walk their way to the equilibrium.

The same applies in the n-player version. With exact balanced reciprocity – each player begins with random estimates of other players' abilities, interacts with a random sample of other players, witnesses a random subsample of interactions, and revises reputations accordingly – benefits and reputations do not converge on the distributional norm (Fig. S1; compare Almost Balanced Reciprocity, Fig. 2).

Similar reputational dynamics arise with page-ranking algorithms, where web pages are ranked based on recursively defined scores: every page passes on ("pays") some of its score to the pages it links to. If each page transmits 100% of its score this way, the algorithm doesn't settle on a stable ranking. Convergence happens if each page transfers a fraction of its assigned score to randomly selected pages – so some pages get a somewhat higher score than what they "pay" for by attracting links. (For Google's PageRank the fraction is .15) [9].

To calculate a surface for Almost Balanced Reciprocity, we begin with a set of points satisfying $b^B[x,y] = 1.02 \cdot b^B[y,x] = \tau^{-\frac{1-\tau}{\tau}} x$ at $y = 5$ and $0 \le x \le 5$, then calculate values of $b^B[x,y]$ for successively smaller values of y down to 0 satisfying a finite difference version of Condition 3.

6 Generalized Reciprocity

The idea behind generalized reciprocity is to maximize group benefits, subject to the honest advertising constraints. Here I consider a modified version of Conditions 3 and 4, where players observe not just the benefits other players provide, but also the costs they incur. In this case, a distributional norm can be built on the assumption that j's reputation depends not just on the benefits, b_{ij}, she produces for various i, but on her net benefits, $b_{ij} - c_{ji}$. (Note that the donor net benefit, $b_{ij} - c_{ji}$, is different from the recipient net benefit, $b_{ij} - c_{ij}$, featured in Conditions 1 and 2 and Fig. 1.) This allows for a more generous distributional norm. If j tries to impersonate a weaker player with ability y, her costs go down from $\alpha_j^{-\frac{1-\tau}{\tau}} \sum_i b^A[\alpha_i, \alpha_j]^{1/\tau}$ to $\alpha_j^{-\frac{1-\tau}{\tau}} \sum_i b^A[\alpha_i, y]^{1/\tau}$. But other players k, rather than rewarding j as if she really had ability y, i.e. giving her $\sum_k b^A[y, \alpha_k]$, can see that she is producing benefits for other players on the cheap, and reduce the benefits they give her by (up to) $y^{-\frac{1-\tau}{\tau}} \sum_i b^A[s_i, y]^{1/\tau} - \alpha_j^{-\frac{1-\tau}{\tau}} \sum_i b^A[s_i, y]^{1/\tau}$, the difference between costs for a player with ability y, and costs for j, which are lower. This works if other players can use knowledge of j's benefits *and* costs to calculate α_j, according to $\alpha_j^{1-\tau} = \sum_i b_{ij} / \sum_i c_{ji}^{\tau}$.

A really generous distributional norm built around observing other players' net benefits could equalize net benefits for all players, regardless of ability. At this honest advertising limit, however, the norm is no longer stable, because the penalty for impersonating a weaker or stronger player disappears – and close to the limit, the penalty is slight. (Note that at this honest advertising limit, players are still not as generous as under pure altruism.) An intermediate norm, based on a combination of observed benefits and observed net benefits, is more workable.

$$\begin{aligned}
&\sum_k b^G[\alpha_j, \alpha_k] - \alpha_j^{-\frac{1-\tau}{\tau}} \sum_i b^G[\alpha_i, \alpha_j]^{1/\tau} \\
&\quad > (1-\gamma)\left(\sum_k b^G[y, \alpha_k] - \alpha_j^{-\frac{1-\tau}{\tau}} \sum_i b^G[\alpha_i, y]^{1/\tau}\right) \\
&\quad\quad + \gamma\left(\sum_k b^G[y, \alpha_k] - y^{-\frac{1-\tau}{\tau}} \sum_i b^G[\alpha_i, y]^{1/\tau}\right) \qquad (7)
\end{aligned}$$

for $y \neq \alpha_j$ and $0 < \gamma \ll 1$. (There is a continuous version of this condition, analogous to Condition 6. We skip it here.) With $\gamma = 0$, we recover the original anti-impersonation constraint, Condition 3. As γ approaches 1, honest advertising breaks down. For the version of generalized reciprocity considered here, we set $\gamma = .5$.

To calculate a surface for Generalized Reciprocity, we maximize total net benefits

$$\sum \left(b^G[y,x] - y^{-\frac{1-\tau}{\tau}} b^G[x,y]\right) \tag{8}$$

for a closely spaced grid of points within the region $\{0,2\} \times \{0,2\}$ subject to a finite differences version of Condition 7.

A revised version of the implicit definition of reputation, s_j, attending to both benefits and net benefits, is given by:

$$0 = \sum_i \Upsilon_{ij} \cdot \omega^{\Upsilon_{ij}} \tag{9}$$

where

$$\Upsilon_{ij} = (1-\gamma)\left(b_{ij}^{1/\tau} - b^G[s_i,s_j]^{1/\tau}\right) + \gamma \cdot \alpha_j^{\frac{1-\tau}{\tau}}\left(c_{ji} - c^G[s_j,s_i]\right) \tag{10}$$

and

$$c^G[s_j,s_i] = s_j^{-\frac{1-\tau}{\tau}} b^G[s_i,s_j]^{1/\tau} \tag{11}$$

which reduces to Condition 4 for $\gamma = 0$.

7 Finding the norm function b^W for a compromise weight w_0

Here I show how to construct a stable distributional norm W which is a weighted mean of two stable distributional norms – in this case B and G. Let the summed net benefit for each player j be equal to w_0 times her summed net benefit under norm B plus $1 - w_0$ times her summed net benefit under norm G, with $0 \leq w_0 \leq 1$. (This is the left-hand side of Condition 13 below.) Because B and G both provide maximum net benefits to j when each j honestly advertises her ability, their linear combination in norm W does so too.

For a norm W corresponding to a given w_0, we need to figure out what $b^W[x,y]$ looks like. To begin with, let

$$b_{ij} = \left(w_j b^B[\alpha_i,\alpha_j]^{1/\tau} + (1-w_j) b^G[\alpha_i,\alpha_j]^{1/\tau}\right)^\tau \tag{12}$$

where w_j and $1 - w_j$ (which vary with α_j) govern how player j weights the costs of following Rule B and Rule G. We can then solve for w_j satisfying

$$\begin{aligned} & w_0 \sum_i \left(b^B[\alpha_j,\alpha_i] - \alpha_j{}^{-\frac{1-\tau}{\tau}} b^B[\alpha_i,\alpha_j]^{1/\tau}\right) \\ & \qquad + (1-w_0)\sum_i \left(b^G[\alpha_j,\alpha_i] - \alpha_j{}^{-\frac{1-\tau}{\tau}} b^G[\alpha_i,\alpha_j]^{1/\tau}\right) \\ = & \sum_k \left(w_k b^B[\alpha_j,\alpha_k]^{1/\tau} + (1-w_k) b^G[\alpha_j,\alpha_k]^{1/\tau}\right)^\tau \\ & \qquad - \alpha_j{}^{-\frac{1-\tau}{\tau}} \sum_i \left(w_j b^B[\alpha_i,\alpha_j]^{1/\tau} + (1-w_j) b^G[\alpha_i,\alpha_j]^{1/\tau}\right) \end{aligned} \tag{13}$$

simultaneously for j from 1 to n. Finally, we can fit a curve through the various w_j's and α_j's to construct a continuous function $w[w_0, y]$ for which $w[w_0, \alpha_j] = w_j$ to get

$$b^W[x,y] = \left(w[w_0,y] \cdot b^B[x,y]^{1/\tau} + (1 - w[w_0,y]) \cdot b^G[x,y]^{1/\tau}\right)^\tau \tag{14}$$

thus satisfying Condition 12 for all j (Fig. S2).

We can also define a third type of reputation for each player j, s_j^W, from a linear combination of Conditions 4 and 9.

8 A rule of thumb for a W that beats B and G

For a mixed group containing type B, type G, and type W players, with type B and G players playing by Rules B and G without compromise, we want to find w_0 such that type W players following w_0 do better than either of the other types. A rule of thumb that generally achieves this (but which might be improved on) is generated by finding w_0 for which $b^W[s_i^W, \alpha_j]^{1/\tau}$ gives a least-squares best fit to $b_{ij}^{1/\tau}$, i.e. w_0 corresponding to

$$\min \left[\sum_{j \in B} \sum_i \left(b^W[s_i^W, \alpha_j]^{1/\tau} - b^B[s_i^B, \alpha_j]^{1/\tau}\right)^2 + \sum_{j \in G} \sum_i \left(b^W[s_i^W, \alpha_j]^{1/\tau} - b^G[s_i^G, \alpha_j]^{1/\tau}]\right)^2 \right] \tag{15}$$

(The additional $b_{ij}^{1/\tau} = b^W[s_i^W, \alpha_j]^{1/\tau}$ terms for $j \in W$ cancel out.) This amounts to letting both type B and G "vote" on an outcome, with player j with greater $b_{ij}^{1/\tau}$'s having more of a vote.

In a group with only types B and W (or only types G and W), type W plays according to norm B (or norm G). In the chameleon-on-a-mirror special case of a group with only type W, Condition 15 assigns no value to w_0. For this case, I assign the same net benefits as for a group with $n_B = n_G = 1$; results are similar when net benefits are assigned using random w_0's.

A more rigorous approach would be to write a system of equations such that each type W player j adopts a w_j which maximizes her summed net benefits, given that other Type W players do the same and type B and G players play by Rules B and G. (The w_j's in this case are generally not consistent with a single w_0 for the group.) I gave up on this approach, in favor of finding a good-enough rule of thumb by trial and error, for several reasons. Computing numerical solutions for the corresponding system of simultaneous differential equations was very slow. And when a large fraction of the population was Type W, the system often didn't converge at all on a solution.

9 Further issues

Some assumptions that might be modified in future work:

Population structure and group selection. This article puts social dynamics in the foreground, and population dynamics in the background, so I adopt the simplest approach to within- and between-group selection and relatedness, equating average context-dependent individual net benefits for each type with fitness increments, in an infinite population of groups with no spatial structure, and a coefficient of relatedness given exogenously. Results might change with an explicit model of group formation, extinction, and replacement; intergroup migration; and/or spatial structure. For

example, group replacement through intergroup competition [10, 11] would probably intensify selection for generalized reciprocity.

Despotic distributions and fratricide. Here I consider just two distributional norms, and a one-dimensional family of intermediate types. But there are other possibilities. Consider the family of despotic norms: norm D_y, if adopted by the whole group, maximizes net benefits for a player with ability y, subject to the honest advertising constraints. In a group in which all other players adapt to any prevailing norm(s) without pushing any norm of their own, a despotic moralist j who plays D_y with $y = \alpha_j$ will do exceptionally well. Other players i with $\alpha_i \neq y$ will do badly, but each will do even worse if she violates the norm. (This is the n-person version of the finding that, in an Iterated Prisoner's Dilemma game, an extortionate player who plays a "zero-determinant" strategy can take advantage of a more adaptable player [12].) This kind of despotic moralizing is fratricidal if pursued by more than one player: if many group members (e.g. a would-be despot and her relatives) promote the various inconsistent D_y's associated with their various abilities, without compromising, all do badly. But more work needs to be done on the vulnerability of compromising players to exploitative norms.

Open groups and reported reputation. Here I assume that players interact in closed face-to-face groups. Each player assesses reputation independently by observing a fraction of all other players' interactions. In a different model, players in open social networks [13–16] might help others they haven't observed directly based on reported reputation [17, 18].

Scaling up. Social mechanisms like segmentation, stratification, and formal institutions can allow enforcement of rules in larger groups, whose component parts could still be face-to-face reputation-based groups. However calculating coefficients of relatedness in large, long-lasting groups is problematic: in such groups, shared genes may reflect a shared history of selection more than shared descent [2]. When organisms are related through large numbers of distant ancestors, the r between individuals at loci influencing social behavior may be inconsistent, as a result of selection, with r based on genes identical-by-descent across the whole genome. For this reason, socially enforced nepotism, at least as developed here, does not scale up indefinitely. Different models are needed to explore the evolution of distributional norms on the scale of ethnic groups and larger.

References

1. Bowles S. Group competition, reproductive leveling, and the evolution of human altruism. Science. 2006;304:1569–1572.
2. Jones D. Group nepotism and human kinship. Current Anthropology. 2000;41:779–809.
3. Panchanathan K, Boyd R. Indirect reciprocity can stabilize cooperation without the second-order free rider problem. Nature. 2004;432:499–502.
4. Leimar O, Hammerstein P. Evolution of cooperation through indirect reciprocity. Proceedings of the Royal Society B. 2001;268:745–753.
5. Panchanathan K, Boyd R. A tale of two defectors: the importance of standing for evolution of indirect reciprocity. Journal of Theoretical Biology. 2003;224:115–126.
6. Ohtsuki H, Iwasa Y. The leading eight: social norms that can maintain cooperation by indirect reciprocity. Journal of Theoretical Biology. 2006;239:435–444.

7. Roberts G. Competitive altruism: from reciprocity to the handicap principle. Proceedings of the Royal Society B. 1998;265:427–431.
8. Nowak MA, Sigmund K. Evolution of indirect reciprocity by image scoring. Nature. 1998;393:573–577.
9. Langville AN, Meyer CD. Google's PageRank and Beyond: The Science of Search Engine Rankings. Princeton University Press; 2012.
10. Choi JK, Bowles S. The coevolution of parochial altruism and war. Science. 2007;318:636–640.
11. Bowles S. Did warfare among ancestral hunter-gatherers affect the evolution of human social behaviors? Science. 2009;2009:1293–1298.
12. Press W, Dyson F. Iterated Prisoner's Dilemma contains strategies that dominate any evolutionary opponent. Proceedings of the National Academy of Sciences. 2012;109:10409–10413.
13. Du WB, Cao XB, Hu MB, Wang WX. Asymmetric cost in snow drift game on scale-free networks. EPL. 2009;87(6004):1–6.
14. Zhen Wang MJ Satoshi Kokubo, Tanimoto J. Universal scaling for the dilemma strength in evolutionary games. Physics of Life reviews. 2015 September;14:1–30.
15. Xia C, Miao Q, Wang J, Ding S. Evolution of cooperation in the traveler's dilemma game on two coupled lattices. Applied Mathematics and Computation. 2014 1 November;246.
16. Xia CY, Meng XK, Wang Z. Heterogeneous coupling between interdependent lattices promotes cooperation in the Prisoner's Dilemma Game. PLoS One. 2015;10(6):e0129542.
17. Mohtashemi M, Mui L. Evolution of indirect reciprocity by social information: the role of trust and reputation in evolution of altruism. Journal of Theoretical Biology. 2003;223:523–531.
18. Sommerfeld RD, Krambeck HJ, Semman D, Milinski M. Gossip as an alternative for direct observation in games of indirect reciprocity. Proceedings of the National Academy of Sciences. 2007;104:17435–17440.

10 S1 and S2 Figs

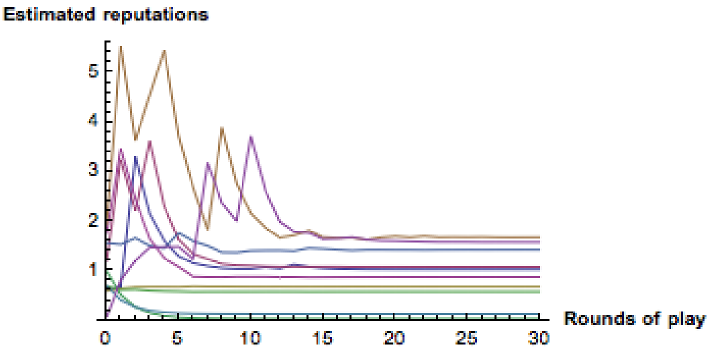

Figure 1. *Not reaching equilibrium with exact balanced reciprocity.* As in Fig. 2, but with exact (not almost) balanced reciprocity. Estimated reputations do not converge on α_j. (Many estimated reputations are wildly exaggerated, but adding a ceiling on estimates does not result in convergence.)

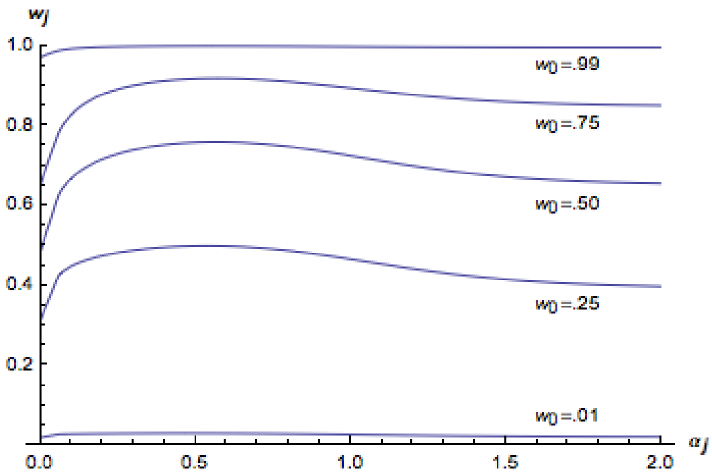

Figure 2. *Weights for a compromise norm.* For different values of w_0, this shows how a player with ability α_j should weight her costs between b^B and b^G to achieve a compromise distributional norm satisfying Condition 13 for stability.
