# Supplementary figures and images for: Socially Enforced Nepotism: How Norms and Reputation Can Amplify Kin Altruism
Source: PLoS One. 2016 Jun 15;11(6):e0155596. doi: 10.1371/journal.pone.0155596 (PMC4909296; doi:10.1371/journal.pone.0155596)

# Estimated reputations

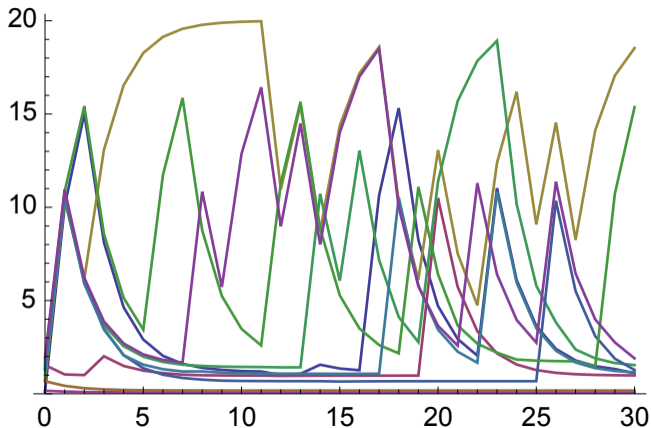

Rounds of play

Supplement: S1 Fig — As in Fig 2, but with exact (not almost) balanced reciprocity. Estimated reputations do not converge on αj. (Many estimated reputations are wildly exaggerated, but adding a ceiling on estimates does not result in convergence.) (PDF) [file pone.0155596.s002.pdf]

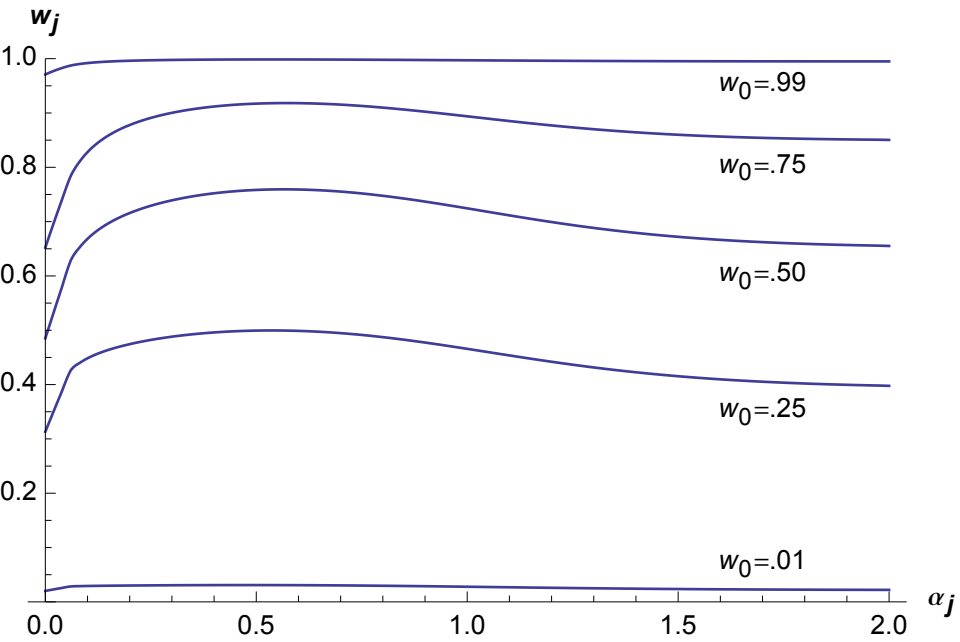

Supplement: S2 Fig — For different values of w0, this shows how a player with ability αj should weight her costs between bB and bG to achieve a compromise distributional norm satisfying Condition 13 for stability (See S1 File). (PDF) [file pone.0155596.s003.pdf]
